# Length of Barrett’s esophagus in the presence of low-grade dysplasia, high-grade dysplasia, and adenocarcinoma

**DOI:** 10.1007/s00464-020-07950-5

**Published:** 2020-09-02

**Authors:** Jenifer Barrie, Fady Yanni, Mohamed Sherif, Asha K. Dube, Anand P. Tamhankar

**Affiliations:** 1 Department of Upper Gastrointestinal Surgery, Sheffield Teaching Hospitals NHS Foundation Trust, Sheffield, UK; 2 Department of Histopathology, Sheffield Teaching Hospitals NHS Foundation Trust, Sheffield, UK; 3 Academic Unit of Surgery, University of Sheffield, Northern General Hospital, Herries Road, Sheffield, S5 7AU South Yorkshire UK

## Abstract

**Introduction:**

The identification and follow-up of ultra-short Barrett’s esophagus (BE) is controversial. BE surveillance guidelines emphasize mainly on long-segment BE. However, in practice a substantial proportion of esophageal adenocarcinoma (EAC) are found close to the gastro-esophageal junction (GEJ). Our study aims to chart the length of BE when low-grade dysplasia (LGD), high-grade dysplasia (HGD) and EAC arise in BE.

**Methods:**

Endoscopic findings from all cases with a diagnosis of LGD and HGD in BE between June 2014 and June 2019, and 100 consecutive cases of EAC diagnosed between June 2018 and August 2019, were reviewed. Additionally, 438 consecutive gastroscopies were reviewed to identify 100 cases of non-dysplastic BE.

**Results:**

99 cases of LGD and 61 cases of HGD were reviewed. LGD and HGD when diagnosed, was located in BE ≤ 1 cm in 20% and 18% cases, respectively. LGD and HGD when diagnosed, was located in BE ≤ 3 cm in 48.5% and 40.9% cases, respectively. LGD and HGD when diagnosed in BE ≤ 3 cm was found at index endoscopy in 67% and 42% cases, respectively. Of the 100 cases of EAC, only 23 had concurrent visible BE, with BE higher than the level of EAC in seven. EAC when found, had its proximal extent ≤ 1 cm from GEJ in 22% and ≤ 3 cm from GEJ in 40% cases. Of the 100 non-dysplastic BE, 53% were ≤ 1 cm and 78% were ≤ 3 cm long.

**Conclusion:**

Almost 20% of all dysplasia in BE occurs in BE < 1 cm. Over 40% occurs in BE < 3 cm. Similarly, 20% of EAC occurs within 1 cm of GEJ and 40% occur within 3 cm. A majority of dysplasia diagnosed within 3 cm of the GEJ is found on index endoscopy. We propose that all lengths of columnar lined epithelium above the GEJ are recognized as BE and subjected to a thorough biopsy protocol.

The prevalence and incidence of esophageal adenocarcinoma (EAC) is increasing in the western world [1, 2]. Risk factors for EAC are Barrett’s esophagus (BE) and longstanding symptomatic gastro-esophageal reflux disease (GERD) [1, 3]. It is accepted that BE progression follows the classic cellular sequence of BE to low-grade dysplasia (LGD) to high-grade dysplasia (HGD) to EAC [4–6]. As a result, it is established practice to offer surveillance once BE is diagnosed.

Several studies have shown that BE progression rate is generally very low but is higher as the length of BE increases [7–9]. Short-segment BE (SSBE) defined as BE ≤ 3 cm is considered to have lower risk of progression as against long-segment BE (LSBE) defined as BE > 3 cm. [4, 10, 11]. It is also accepted that BE diagnosed EAC has better prognosis and survival advantage [12–14].

This has led to authoritative guidelines both by the American College of Gastroenterologists (ACG) and British Society of Gastroenterologists (BSG) recommending BE surveillance protocols [15, 16]. Guidelines summarized in Table 1 do focus on more regular LSBE surveillance with much infrequent SSBE surveillance. Ultra-short BE (USBE) defined as BE ≤ 1 cm is not even considered for diagnosis or surveillance.

**Table 1:** Current ASG and BSG guidelines on Barrett’s surveillance

ASG guideline		BSG guideline	
BE length	Surveillance interval	BE length	Surveillance interval
< 1 cm	No further surveillance	< 1 cm	No further surveillance
> 1 cm	3–5 years	< 3 cm without intestinal metaplasia	No further surveillance
		< 3 cm with intestinal metaplasia	3–5 years
		> 3 cm	2–3 years

Despite acknowledging that BE is a risk factor for development of EAC and having surveillance programs for several decades, the current BE surveillance detected EAC rate remains very low with only 3–8% of diagnosed EAC have previously known BE [14, 17, 18]. It takes around 200 patients years of BE surveillance to diagnose one EAC, with most patients with BE dying of other causes [19–21]. The financial burden of BE is also high for health care systems and individuals [22, 23]. Furthermore, the survival advantage for BE surveillance diagnosed EAC is often questioned. In their case–control study, Corley et al. found that BE surveillance did not lead to improved EAC outcomes [24]. Better outcomes in EAC diagnosed by BE surveillance could also be due to lead time and length time bias [25]. As a result, unless the EAC pick-up rate from BE surveillance is improved radically, BE surveillance cannot be considered very effective.

We postulate that the focus on LSBE surveillance as against SSBE and disregarding USBE from BE diagnosis may be partly responsible for poor EAC pick-up rate on BE surveillance. If longer length of BE is an important predictor of progression then we should either find EAC more frequently higher up and away from the gastro-oesophageal junction (GEJ) within a LSBE or alternatively simply a long EAC involving the entire LSBE; or a focal EAC near the GEJ with LSBE above it. However, in practice, a substantial proportion of EAC are found close to the GEJ without co-existent long-segment BE. Our study aims to chart the length of BE when LGD, HGD and EAC arise in BE and to identify the topographic location of EAC and non-dysplastic BE.

## Methods

### Study design

This was a retrospective analysis of a contemporaneously maintained database at a regional upper gastro-intestinal cancer center in the United Kingdom. Patients with a diagnosis of LGD or HGD in BE and EAC were included. Data collection included patient demographics (such as gender and age at first endoscopy diagnosis), histology results, and confirmation of histological diagnosis by 2 histopathologists and presence or absence of intestinal metaplasia. Description of BE was done endoscopically using the Prague classification system that measures the circumferential (C) and maximal (M) extents of BE lengths introduced by the International Working Group for the Classification of Esophagitis (IWGCO) in 2004 [26].

In addition, 438 consecutive gastroscopies by a single endoscopist who accurately recorded columnar lined epithelium above the GEJ were analyzed to identify 100 cases of non-dysplastic BE.

### Three groups of data were analyzed:

*Group 1 (Dysplastic BE)* All cases with a histological diagnosis of LGD and HGD in BE between June 2014 and June 2019. For these patients, their demographic data and endoscopic features were reviewed.

*Group 2 (EAC)* 100 consecutive cases of EAC diagnosed between June 2018 and August 2019. For these patients, their demographic data and specific endoscopic features including exact site of tumor, upper extent of cancer from the GEJ and any associated BE above the level of EAC were reviewed.

*Group 3 (Non-dysplastic BE)* 100 consecutive cases of non-dysplastic BE between June 2018 and August 2019. For these patients, their demographic data and endoscopic features were reviewed.

### Sample size

Dysplasia in BE is not very common. To maximize the number of cases in the dysplastic BE group, we included all consecutive cases recorded as LGD or HGD in BE from our histopathology database over the preceding five years at the start of our study. After applying the exclusion criteria, discussed below, a study sample of 160 cases on dysplastic BE cases were analyzed. A sample size of 100 consecutive EAC and 100 non-dysplastic BE were deemed adequate to represent the more common conditions of EAC and BE.

### Definitions

**Fig. 1:**
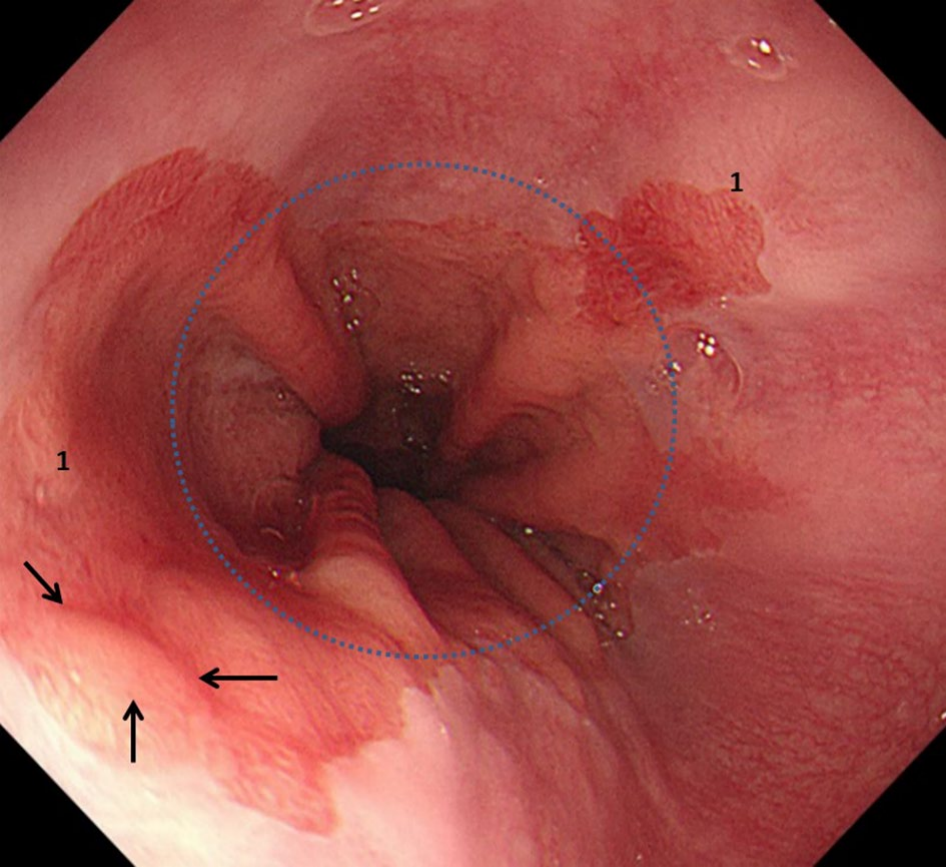
Barrett’s esophagus was classed as USBE (1) when the squamo-columnar junction was < 1 cm above the gastro-esophageal junction or the level of the highest gastric mucosal fold (blue dotted circle). Incidentally, in this case HGD was found in the nodule (arrows)

For the purposes of this study, BE was defined, according to the BSG guidance, as an esophagus in which any portion of the normal distal squamous epithelial lining had been replaced by metaplastic columnar epithelium, which was clearly visible endoscopically above the GEJ and confirmed histopathologically from esophageal biopsies [16]. A histological diagnosis was performed as per minimum reporting standards in the BSG guideline. Therefore the presence of intestinal mucosa was not necessary for BE in our study. LGD or HGD was identified by two histopathologists or confirmed at Multi Disciplinary Team (MDT) review where a two histopathology review was not achieved. Other definitions are as follows:

USBE was defined as up to 1 cm of columnar lined mucosa above the gastric mucosal folds (see Fig. 1).

**Fig. 2:**
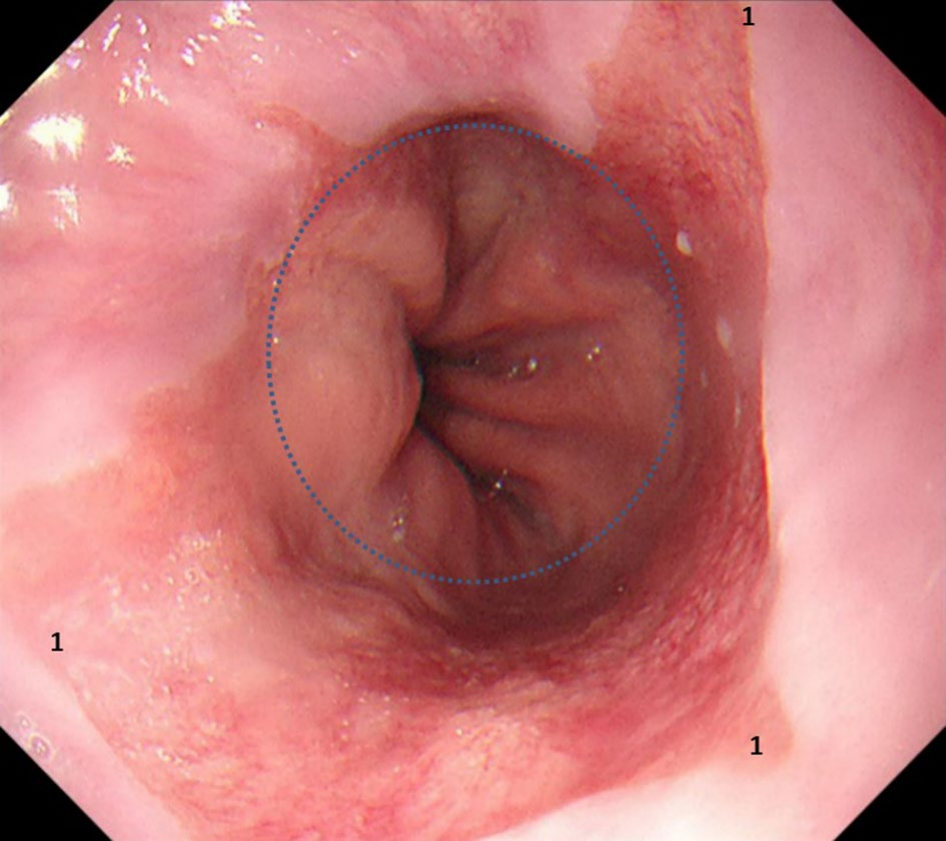
Barrett’s esophagus was classed as SSBE (1) when the squamo-columnar junction was ≥ 1 cm to < 3 cm above the gastro-esophageal junction or the level of the highest gastric mucosal fold (blue dotted circle). Incidentally, in this case LGD was found with no visible lesion

SSBE is defined as ≥ 1 cm to < 3 cm Barrett’s esophagus (see Fig. 2).

**Fig. 3:**
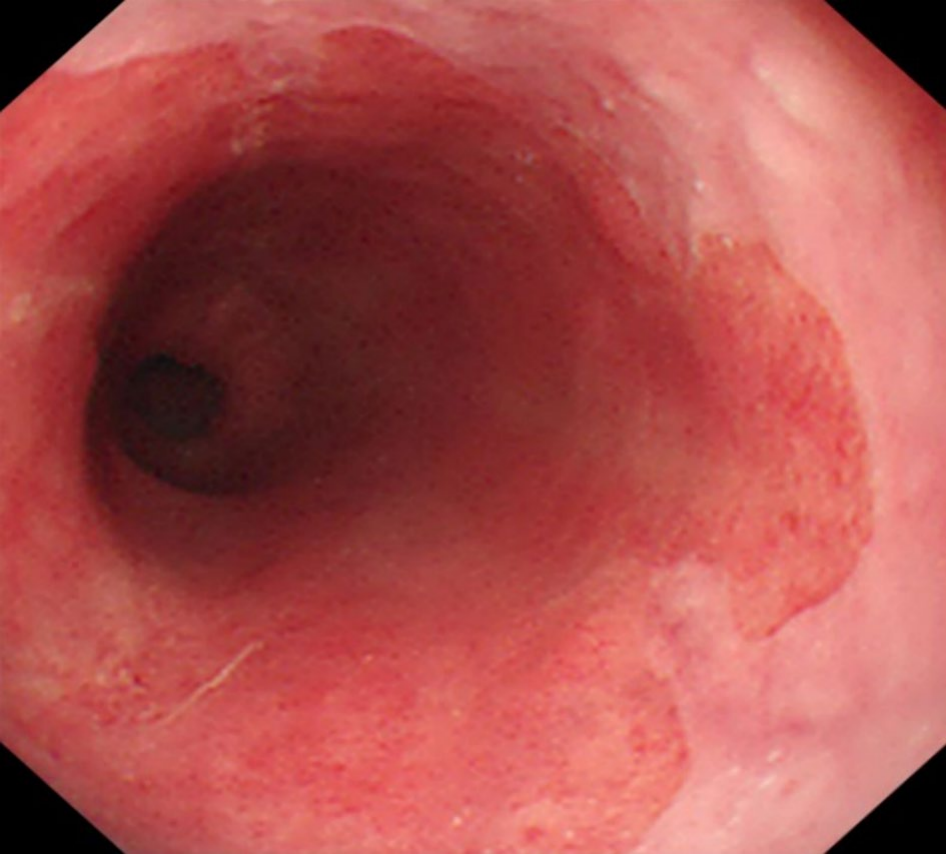
Barrett’s esophagus was classed as LSBE when the squamo-columnar junction was > 3 cm above the gastro-esophageal junction

LSBE is defined as ≥ 3 cm Barrett’s esophagus (see Fig. 3).

### Exclusion criteria

*For Group 1 (Dysplastic BE)* Any patient who had squamous dysplasia, indeterminate dysplasia or those who had previous history of endoscopic ablative or resectional therapy were excluded. Data for patients with a diagnosis of dysplasia more than once in the study period were recorded from the initial diagnostic episode.

*For Group 2 (EAC)* Patients with Siewert type III GEJ tumors were excluded.

**Fig. 4:**
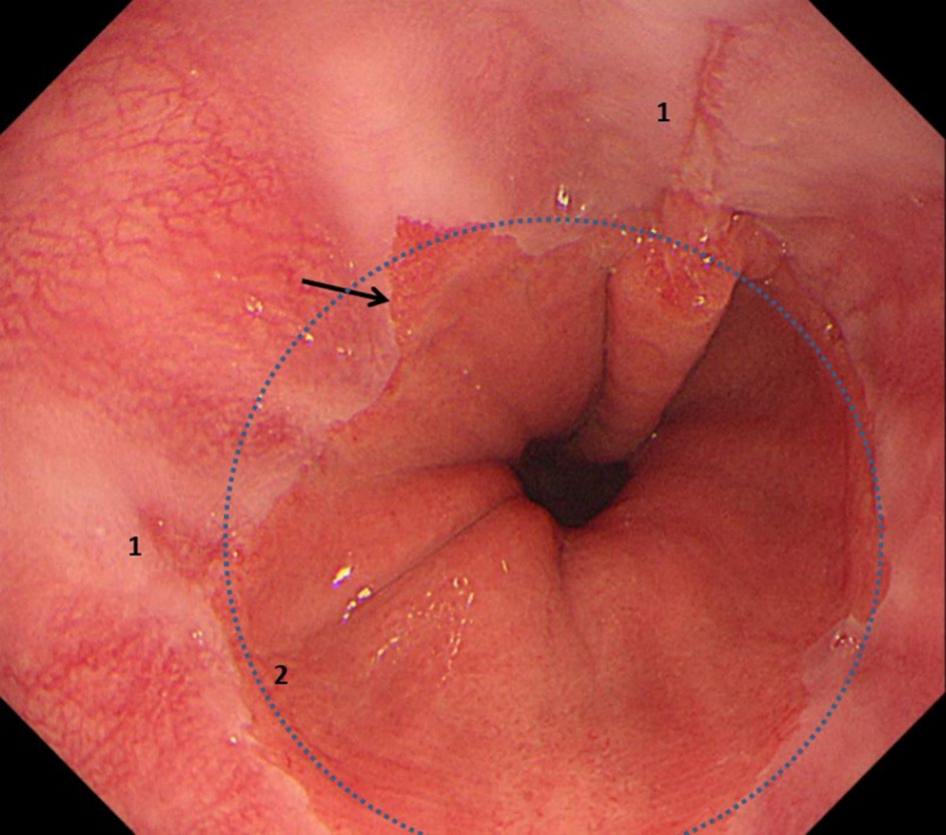
Squamo-columnar junction was defined as ‘*irregular z-line*’ (bold arrow) rather than Barrett’s esophagus, when was present with inflammation (1) and mostly within the level (blue dotted circle) of the highest gastric mucosal fold (2)

*For Group 3 (non-dysplastic BE)* Patients with irregular *z-line* (squamo-columnar junction) were excluded (see Fig. 4).

### Ethics and consent

Data analyzed in this study was used from a contemporaneously maintained database from Histopathology and Endoscopy units at our institute. Data review was approved by local Clinical Effectiveness Unit (CEU project registration number: 9341). No patient identifying information was recorded and patient consent was not required for data review as per CEU guidelines.

## Results

### Group 1

This group comprised 160 patients. 99 cases of LGD and 61 cases of HGD were identified. Median age was 70. 126 were male (79%). 118 (74%) of cases were reviewed by 2 pathologists. All cases having a diagnosis of LGD for the second time or after initial diagnosis of HGD were reviewed in a MDT meeting. Median circumferential BE was 2 cm and median maximal extension was 4 cm. Background intestinal metaplasia was confirmed histologically in 142 (89%) of these cases.

LGD and HGD when diagnosed, was located in BE ≤ 1 cm in 20 cases (20%) and 11 cases (18%), respectively. LGD and HGD when diagnosed, was located in BE ≤ 3 cm in 48 cases (48.5%) and 25 (40.9%) cases, respectively. In 30 cases (19%) either LGD or HGD was present < 1 cm from the GEJ. Table 2 shows a detailed comparison of results between the LGD and HGD groups.

**Table 2:** Demographics, pathology and position of LGD and HGD

	LGD	HGD
Number of cases	99	61
Gender	Male = 79 (79.8%)	Male = 47 (77%)
	Female = 20 (20.2%)	Female = 14 (23%)
Median age	69	70
2 pathologist reporting	67 (67.7%)	51 (83.6%)
Median circumferential BE	2 cm	2 cm
Background intestinal metaplasia	88 (88.8%)	54 (88.5%)
Median maximal BE extensions	4 cm	4 cm
Length of BE from GEJ		
< 1 cm	20 (20.2%)	11 (18%)
< 3 cm (includes < 1 cm)	48 (48.5%)	25 (40.9%)
> 3 cm	51 (51.5%)	36 (59%)

LGD and HGD when diagnosed in BE ≤ 3 cm, was found at index endoscopy in 67% and 42% cases, respectively. The rest of the cases were diagnosed while on BE surveillance. Of all the cases of LGD in BE ≤ 3 cm found at index endoscopy, 65% had no visible lesions in the BE segment, whereas the rest had ulcer/inflammation (19%) or nodules (16%). In these cases of LGD (in BE ≤ 3 cm), the commonest indications for index endoscopy were reflux symptoms (39%), dysphagia (32%) and anemia or gastro-intestinal (GI) bleeding (19%). Of all cases of HGD in BE ≤ 3 cm found at index endoscopy, 50% had no visible lesions in the BE segment, whereas nodules were seen in 40% and ulcer in 10%. In these cases of HGD (in BE ≤ 3 cm), the indications for index endoscopy were dysphagia (50%), reflux symptoms (30%) and anemia (20%).

The proportion of LGD and HGD diagnosed at index endoscopy was even higher in cases when BE was ≤ 1 cm, with 70% and 67% of cases being diagnosed at index endoscopy, respectively. In these cases of LGD and HGD diagnosed at index endoscopy, when BE was ≤ 1 cm long, 64% and 73%, respectively, had no visible lesions in the BE segment.

### Group 2

Group 2 comprised 100 consecutive cases of EA diagnosed between June 2018 and August 2019. In this category, 78 (78%) were males. Median age at diagnosis was 70 years. Only 23 cases (23%) had concurrent visible BE. If no BE was visible, it was acknowledged that the EAC had completely overgrown into the existing Barrett’s epithelium.

In 22 (22%) cases EAC was reported to have a proximal extent ≤ 1 cm from GEJ. 18 cases (18%) were detected between 1 and 3 cm from the GEJ, and the majority of cases (60%) were detected > 3 cm above GEJ.

Of the 100 cases of EAC, BE was visible above the level of tumor in 7 cases. When BE was visible concurrently with EAC: it was recorded ≤ 1 cm from the GEJ in 6 cases; ≤ 3 cm from the GEJ in 14 cases; and > 3 cm from the GEJ in 9 cases.

### Group 3

This group consisted of 100 cases of non-dysplastic BE between June 2018 and August 2019. 71 patients were male. Median age was 68 years. In 53 cases (53%) BE was detected within 1 cm and 78 cases (78%) were ≤ 3 cm of the GEJ. 22 cases (22%) were reported as > 3 cm (LSBE).

## Discussion

Our study focussed on measuring the length of BE when LGD, HGD and EAC were diagnosed. Our results show that a substantial proportion of the diagnosed LGD and HGD are located in short-segment BE, with around one-fifth in ≤ 1 cm BE. Similarly around one-fifth of EAC occur within 1 cm of the GEJ without adjacent long-segment BE. When accurately identified, by reviewing 438 consecutive gastroscopies, over 50% of BE is ≤ 1 cm of the GEJ. This would lead us to postulate that current system of Barrett’s surveillance will not pick-up a proportion of EAC and dysplasia as columnar lined epithelium ≤ 1 cm is disregarded from diagnosis and surveillance.

Though LSBE is considered as a risk factor for progression, multiple studies show SSBE risk is statistically similar to LSBE [7, 10, 27]. ACG and BSG accept that BE surveillance guidelines are based on poor quality evidence [16, 23]. If most studies do not include USBE in the diagnosis and surveillance of BE, the established literature would invariably show a bias towards LSBE which is easy to identify and study. LSBE and SSBE are arbitrarily defined by a length of 3 cm from GEJ. It is well recognized that there is a substantial inter-observer variation in the measurement of BE length [26]. Alvarez Herrero et al. have shown that BE length may vary by 1–2 cm between two endoscopic records [28]. Similar observer variability in recording BE length has been shown previously [29, 30]. This means that there is the potential for a 2 cm BE to be called USBE and ignored from surveillance, and a 2–3 cm BE called LSBE, falsely increasing the perceived risk from LSBE. We believe that stratifying BE risk based on length < or > than 3 cm is arbitrary, prone to observer error and clearly not working. Furthermore, the established system for biopsy in BE, requires 4-quadrant biopsies to be taken at every 1–2 cm length. If observer error is recognized in establishing the length of BE, it would adversely affect the number of biopsies taken in shorter lengths of BE.

Various studies have reported and acknowledged the dysplasia risk associated with SSBE and USBE [31–33]. Their results demonstrate a dysplasia risk of 8 to 12% in patients with SSBE. Pohl et al. in their elaborate study in the German population, demonstrated that patients diagnosed with early (T1) EAC had SSBE in 24% cases and USBE in 20% (overall BE ≤ 3 cm in 44% cases) [34]. Unfortunately, such studies haven’t impacted in the BE surveillance guidelines as the overall prevalence of SSBE and USBE in general population is quite high making surveillance financially unviable for SSBE and USBE. As such the prevalence of BE in asymptomatic male veterans older than 50 years of age is as high as 25% with 70% of them ≤ 3 cm long [35]. This is not dissimilar to our study where 23% of 438 consecutive endoscopies had BE with 78% being ≤ 3 cm long. This means that while it is possible to admit the dysplasia and EAC risk of USBE and SSBE, it does not bode well for a surveillance program as it will need thousands of endoscopies to diagnose one EAC. Looking at the population prevalence of BE, Pohl et al. concluded that over 3000 patients with SSBE and over 12,000 patients with USBE would need endoscopic assessment to diagnose one EAC in each group [34]. Though Pohl and colleagues only looked at T1 EAC, we do not expect the numbers to reduce drastically if more advanced cancers were also included in their study.

Based on current literature discussed above, we must recognize that the measurement of BE length may be inaccurate; SSBE and USBE both have a risk of progressing to dysplasia; and surveillance of all cases of BE is logistically impossible. A different strategy is necessary to identify patients likely to progress from BE to EAC.

In our study, a vast majority of LGD and HGD, particularly when BE was ≤ 3 cm long, were diagnosed at index endoscopy rather than on surveillance. On sub-analysis of the USBE group, the proportion of LGD and HGD diagnosed on index endoscopy increased further to 70% and 67% cases, respectively. Of patients diagnosed with LGD and HGD on index endoscopy, when BE was ≤ 1 cm long, 64% and 73%, respectively, had no visible lesions in the BE segment. This data emphasizes the need for more focussed biopsies of USBE segment when seen rather than disregarding it from the diagnosis of BE. Similar findings were noted by two other prospective cohort studies which reported a dysplasia rate at index endoscopy of over 8% for patients with a new diagnosis of BE ≤ 3 cm [32, 33].

Our results have demonstrated that a substantial proportion of dysplasia and EAC occur within USBE and SSBE. This highlights the need for recognizing all lengths of columnar lined mucosa as BE when seen at endoscopy. Once BE is identified multiple accurate directed biopsies should be taken to exclude baseline dysplasia at index endoscopy. We hypothesize that BE surveillance detects a very small proportion of EAC due to the lead time bias with only the slow growing tumors being picked on surveillance. It takes several years or decades to develop a truly long LSBE. Inherently, such slow growing LSBE is stable and hence dysplasia or EAC occurring in it is more likely to be picked up on surveillance. In contrast, the majority of EAC not picked on surveillance, perhaps occurs in unstable USBE and SSBE, which converts into EAC rapidly without ever having a chance to develop into true LSBE. We propose that every BE, irrespective of its length, be thoroughly biopsied at every endoscopic opportunity, whether or not they are on surveillance.

Our study was a retrospective observational study with a starting point of dysplasia in BE. This allowed us to review a large number of LGD and HGD cases for their endoscopic features. To assess true dysplasia risk, one would ideally require a large prospective cohort study looking at various lengths of BE on a longitudinal follow-up. While this was outside the aim and scope of our research, we have managed to demonstrate that 46% of the diagnosed dysplasia (LGD and HGD) occurs within 3 cm from the GEJ. A prospective longitudinal study could only increase that proportion due to even better pick-up rate.

## Conclusion

Almost 20% of all dysplasia in BE and EAC occur within a centimeter of the GEJ. Over 40% occur within 3 cm from the GEJ. A majority of dysplasia diagnosed within 3 cm of the GEJ is found on index endoscopy. We propose that all lengths of columnar lined epithelium above the GEJ are recognized as BE and subjected to a thorough biopsy protocol.

## References

[1] Coleman HG, Xie S-H, Lagergren J (2018). The epidemiology of esophageal adenocarcinoma. Gastroenterology.

[2] Kroep S, Lansdorp-Vogelaar I, Rubenstein J, Lemmens V, Van Heijningen E, Aragonés N (2014). Comparing trends in esophageal adenocarcinoma incidence and lifestyle factors between the United States, Spain, and the Netherlands. Am J Gastroenterol.

[3] Maret-Ouda J, Konings P, Lagergren J, Brusselaers N (2016). Antireflux surgery and risk of esophageal adenocarcinoma: a systematic review and meta-analysis. Ann Surg.

[4] Solanky D, Krishnamoorthi R, Crews N, Johnson M, Wang K, Wolfsen H (2019). Barrett esophagus length, nodularity, and low-grade dysplasia are predictive of progression to esophageal adenocarcinoma. J Clin Gastroenterol.

[5] Singh S, Manickam P, Amin AV, Samala N, Schouten LJ, Iyer PG (2014). Incidence of esophageal adenocarcinoma in Barrett's esophagus with low-grade dysplasia: a systematic review and meta-analysis. Gastrointest Endosc..

[6] Duits LC, Phoa KN, Curvers WL, Ten Kate FJ, Meijer GA, Seldenrijk CA (2015). Barrett's oesophagus patients with low-grade dysplasia can be accurately risk-stratified after histological review by an expert pathology panel. Gut.

[7] Desai TK, Krishnan K, Samala N, Singh J, Cluley J, Perla S (2012). The incidence of oesophageal adenocarcinoma in non-dysplastic Barrett's oesophagus: a meta-analysis. Gut.

[8] Hvid-Jensen F, Pedersen L, Drewes AM, Sørensen HT, Funch-Jensen P (2011). Incidence of adenocarcinoma among patients with Barrett's esophagus. N Engl J Med.

[9] Bhat S, Coleman HG, Yousef F, Johnston BT, McManus DT, Gavin AT (2011). Risk of malignant progression in Barrett’s esophagus patients: results from a large population-based study. J Natl Cancer Inst.

[10] Wani S, Falk G, Hall M, Gaddam S, Wang A, Gupta N (2011). Patients with nondysplastic Barrett's esophagus have low risks for developing dysplasia or esophageal adenocarcinoma. Clini Gastroenterol Hepatol.

[11] Wong T, Tian J, Nagar AB (2010). Barrett's surveillance identifies patients with early esophageal adenocarcinoma. Am J Med.

[12] Ding YE, Li Y, He XK, Sun LM (2018). Impact of Barrett's esophagus surveillance on the prognosis of esophageal adenocarcinoma: a meta-analysis. J Digest Dis.

[13] El-Serag HB, Naik AD, Duan Z, Shakhatreh M, Helm A, Pathak A (2016). Surveillance endoscopy is associated with improved outcomes of oesophageal adenocarcinoma detected in patients with Barrett's oesophagus. Gut.

[14] Verbeek RE, Leenders M, Ten Kate FJ, Van Hillegersberg R, Vleggaar FP, Van Baal JW (2014). Surveillance of Barrett's esophagus and mortality from esophageal adenocarcinoma: a population-based cohort study. Am J Gastroenterol.

[15] Shaheen NJ, Falk GW, Iyer PG, Gerson LB (2016). ACG clinical guideline: diagnosis and management of Barrett’s esophagus. Am J Gastroenterol.

[16] Fitzgerald RC, di Pietro M, Ragunath K, Ang Y, Kang J-Y, Watson P (2014). British Society of Gastroenterology guidelines on the diagnosis and management of Barrett's oesophagus. Gut.

[17] Dulai GS, Guha S, Kahn KL, Gornbein J, Weinstein WM (2002). Preoperative prevalence of Barrett's esophagus in esophageal adenocarcinoma: a systematic review. Gastroenterology.

[18] Corley DA, Levin TR, Habel LA, Weiss NS, Buffler PA (2002). Surveillance and survival in Barrett's adenocarcinomas: a population-based study. Gastroenterology.

[19] Gudlaugsdottir S, van Blankenstein M, Dees J, Wilson JP (2001). A majority of patients with Barrett's oesophagus are unlikely to benefit from endoscopic cancer surveillance. Eur J Gastroenterol Hepatol.

[20] Rana P, Johnston D (2000). Incidence of adenocarcinoma and mortality in patients with Barrett’s oesophagus diagnosed between 1976 and 1986: implications for endoscopic surveillance. Dis Esophagus.

[21] Van der Burgh A, Dees J, Hop W, Van Blankenstein M (1996). Oesophageal cancer is an uncommon cause of death in patients with Barrett's oesophagus. Gut.

[22] Cotton CC, Shaheen NJ (2021). Overutilization of endoscopic surveillance in Barrett's esophagus: the perils of too much of a good thing. Am J Gastroenterol..

[23] Association AG (2011). American Gastroenterological Association medical position statement on the management of Barrett's esophagus. Gastroenterology.

[24] Corley DA, Mehtani K, Quesenberry C, Zhao W, De Boer J, Weiss NS (2013). Impact of endoscopic surveillance on mortality from Barrett's esophagus–associated esophageal adenocarcinomas. Gastroenterology.

[25] Tramontano AC, Sheehan DF, Yeh JM, Kong CY, Dowling EC, Rubenstein JH (2017). The impact of a prior diagnosis of Barrett’s esophagus on esophageal adenocarcinoma survival. Am J Gastroenterol.

[26] Sharma P, Dent J, Armstrong D, Bergman JJ, Gossner L, Hoshihara Y (2006). The development and validation of an endoscopic grading system for Barrett’s esophagus: the Prague C & M criteria. Gastroenterology.

[27] Yousef F, Cardwell C, Cantwell MM, Galway K, Johnston BT, Murray L (2008). The incidence of esophageal cancer and high-grade dysplasia in Barrett's esophagus: a systematic review and meta-analysis. Am J Epidemiol.

[28] Herrero LA, Curvers WL, Vanvilsteren FG, Wolfsen H, Ragunath K, Song L-M (2013). Validation of the Prague C&M classification of Barrett’s esophagus in clinical practice. Endoscopy.

[29] Guda NM, Partington S, Vakil N (2004). Inter-and intra-observer variability in the measurement of length at endoscopy: Implications for the measurement of Barrett's esophagus. Gastrointest Endosc.

[30] Dekel R, Wakelin DE, Wendel C, Green C, Sampliner RE, Garewal HS (2003). Progression or regression of Barrett's esophagus—is it all in the eye of the beholder?. Am J Gastroenterol.

[31] Clark GW, Ireland AP, Peters JH, Chandrasoma P, DeMeester TR, Bremner CG (1997). Short-segment Barrett’s esophagus: a prevalent complication of gastroensophageal reflux disease with malignant potential. J Gastrointest Surg.

[32] Sharma P, Morales TG, Bhattacharyya A, Garewal HS, Sampliner RE (1997). Dysplasia in short-segment Barrett's esophagus: a prospective 3-year follow-up. Am J Gastroenterol..

[33] Weston AP, Krmpotich PT, Cherian R, Dixon A, Topalosvki M (1997). Prospective long-term endoscopic and histological follow-up of short segment Barrett's esophagus: comparison with traditional long segment Barrett's esophagus. Am. J. Gastroenterol..

[34] Pohl H, Pech O, Arash H, Stolte M, Manner H, May A (2016). Length of Barrett's oesophagus and cancer risk: implications from a large sample of patients with early oesophageal adenocarcinoma. Gut.

[35] Gerson LB, Shetler K, Triadafilopoulos G (2002). Prevalence of Barrett's esophagus in asymptomatic individuals. Gastroenterology.

